# Digitalisierung der histopathologischen Routinediagnostik: Dos and Don’ts!

**DOI:** 10.1007/s00292-023-01291-5

**Published:** 2024-01-08

**Authors:** Viola Iwuajoku, Anette Haas, Kübra Ekici, Mohammad Zaid Khan, Fabian Stögbauer, Katja Steiger, Carolin Mogler, Peter J. Schüffler

**Affiliations:** 1 Institut für Pathologie, TUM School of Medicine and Health, Technische Universität München, Trogerstraße 18, 81675 München, Deutschland; 2 TUM School of Computational Information and Technology, Technische Universität München, München, Deutschland

**Keywords:** Künstliche Intelligenz, Changemanagement, Computergestützte Bildverarbeitung, Digitale Pathologie, Workflow, Artificial intelligence, Change management, Computer-assisted image processing, Digital pathology, Workflow

## Abstract

Die Digitalisierung des histopathologischen Eingangslabors ist ein wichtiger und entscheidender Schritt in der digitalen Transformation der Pathologie. Digitalisierung ermöglicht zahlreiche neue Optionen wie den Zugang zu großen Datensätzen für KI-gestützte Auswertungen, mobiles Arbeiten und Homeoffice für FachärztInnen sowie eine schnellere und vereinfachte Bereitstellung von Bildern und Daten für Forschungsarbeiten, Konferenzen und Tumorboards. Dennoch bedeutet die Umstellung zu einem vollständig digitalen Workflow auch erheblichen Aufwand im technischen und personellen Bereich und benötigt ein durchdachtes und flexibles Changemanagement, um Reibungsverluste gerade im personellen Bereich möglichst gering zu halten und wertvolles Potenzial talentierter, aber möglicherweise veränderungsscheuer Mitarbeiter nicht zu verlieren. Dieser Artikel fasst die Erfahrungswerte unseres Institutes im Hinblick auf technische und personelle Herausforderungen während der Transformation zur digitalen Pathologie zusammen und bietet einen umfassenden Überblick über potenziell problematische Schnittstellen bei der Umstellung des Routinebetriebes auf einen digitalen Workflow.

## Abstract (en)

The implementation of digital histopathology in the laboratory marks a crucial milestone in the overall digital transformation of pathology. This shift offers a range of new possibilities, including access to extensive datasets for AI-assisted analyses, the flexibility of remote work and home office arrangements for specialists, and the expedited and simplified sharing of images and data for research, conferences, and tumor boards. However, the transition to a fully digital workflow involves significant technological and personnel-related efforts. It necessitates careful and adaptable change management to minimize disruptions, particularly in the personnel domain, and to prevent the loss of valuable potential from employees who may be resistant to change. This article consolidates our institute’s experiences, highlighting technical and personnel-related challenges encountered during the transition to digital pathology. It also presents a comprehensive overview of potential difficulties at various interfaces when converting routine operations to a digital workflow.

Bei der digitalen Pathologie (DP) handelt es sich primär um die Interpretation von Pathologiebildern aus digital gescannten Objektträgern (OT). Zwar ist digitale Pathologie in der Routinediagnostik eine junge Erscheinung – die meisten Pathologielabore arbeiten nach wie vor analog mit Lichtmikroskopen –, allerdings gab es frühe Anfänge der Digitalisierung schon vor Jahrzehnten. In ersten Experimenten wurden Pathologiebilder bereits in den 1960er-Jahren in Echtzeit-Mikroskopie (auch als Telepathologie bezeichnet) zwischen der Logan Airport Medical Station und dem Massachusetts General Hospital in Boston übertragen [1]. Seitdem hat sich diese Technologie zur virtuellen OT-Bildgebung weiterentwickelt, in der mithilfe von OT-Scannern hochauflösende digitale Bilder in Echtzeit erzeugt werden. Digitale Pathologie wurde dadurch erstmals klinisch in der Routine nutzbar [2–4].

## Umstellungsprozess im Labor und bei der Befundung notwendig

Die digitale Pathologie greift sowohl in den Herstellungsprozess der Objektträger (OT) als auch in deren Befundung ein. Um digitale Technologie klinisch nutzen zu können, bedarf es einer sukzessiven Umgestaltung sowohl des histopathologischen Routinelabors als auch des Arztarbeitsplatzes. Neben dem klassischen Nasslabor (Gewebezuschnitt, Einbettung, Schneiden, Färben) ist die Einführung eines elektronischen Laborinformationssystems (LIS, Stichwort „Pathologieinformatik“: Einführung elektronischer Patientenakten und Barcodes zur eindeutigen Kennung von Blöcken und OT) erforderlich. Weiterhin müssen Scanner installiert und an das bestehende LIS angeschlossen werden. Im Labor muss der Arbeitsablauf so angepasst werden, dass hergestellte OT systematisch eingescannt werden. Die Arztarbeitsplätze müssen für das digitale Befunden eingerichtet werden. Schließlich bedarf es der Schulung der ÄrztInnen in der digitalen Befundung, und die gesamte digitale Pathologie inklusive Befundung muss validiert werden, um auch neuere Zertifizierungs- und Akkreditierungsrichtlinien zu erfüllen.

Viele Pathologielabore sind bereits auf einen digitalen Arbeitsablauf umgestiegen und berichten von einer Reihe von Vorteilen [5–10]. So wird von den meisten Laboren über eine steigende Anzahl von Fällen berichtet, die pro Jahr befundet werden können [11, 12]. Zudem sind PathologInnen nicht mehr an einen Laborarbeitsplatz mit Mikroskop gebunden und können auch in speziellen Situationen (Erkrankung der Kinder, COVID-19-Pandemie etc.) remote von zu Hause arbeiten [13–15]. Digitale OT können einfacher und schneller zwischen verschiedenen Standorten geteilt werden und Zweitmeinungen lassen sich schneller einholen [5]. Auch konnte älteres Patientenmaterial von abgeschlossenen Fällen mithilfe digitaler OT leichter und schneller gefunden werden [6].

Digitale Pathologie bringt weitere potenzielle Vorteile mit sich. So wird von einer gesteigerten Effizienz und verbesserten Arbeitsabläufen berichtet [16]. Ziel der digitalen Pathologie ist es auch, PathologInnen in die Lage zu versetzen, mühsame und repetitive Aufgaben effizienter zu lösen und die Genauigkeit der histopathologischen Befunde zu verbessern (z. B. Längenmessungen/Angabe von Infiltrationstiefe und Quantifizierung von immunhistochemischen Färbungen, wie z. B. Mib1/Ki-67-Färbung). Heutzutage haben die meisten PathologInnen ihre Ausbildung unter Einsatz des konventionellen Lichtmikroskops abgeschlossen, daher stellt dieses nach wie vor den Goldstandard in der histopathologischen Diagnostik dar [17, 18]. Um eine optimale digitale Transformation zu erreichen, ist daher eine enge Zusammenarbeit zwischen den PathologInnen und dem technischen Team unerlässlich. Diese Umstellungen im Labor und bei der Befundung machen entsprechende Umstrukturierungen in der IT und im Personalbereich notwendig. Die Umgestaltung betrifft in der Regel langjährig etablierte Betriebsabläufe und greift in zentrale Aspekte der Routine ein. Um diesen Prozess zu vereinfachen, berichten wir im Folgenden über unsere Erfahrungen der digitalen Transformation in unserem Institut.

## Methodik

**Figure 1:**
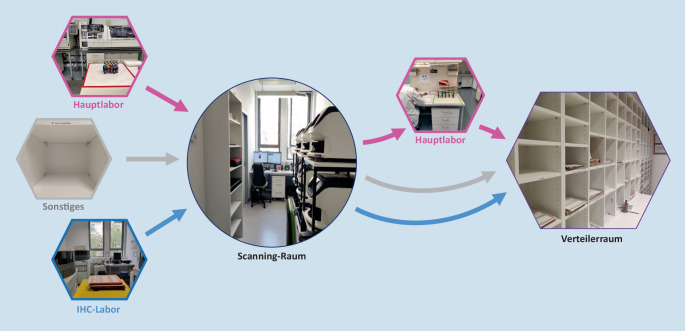

Unser Workflow nach digitaler Transformation ist in Abb. 1 dargestellt. Nachfolgend werden die einzelnen Prozesse in der Umstellung des histopathologischen Routinebetriebes auf diesen Workflow erläutert.

### Voraussetzungen und Ansprüche an die digitale Pathologie

**Figure 2:**
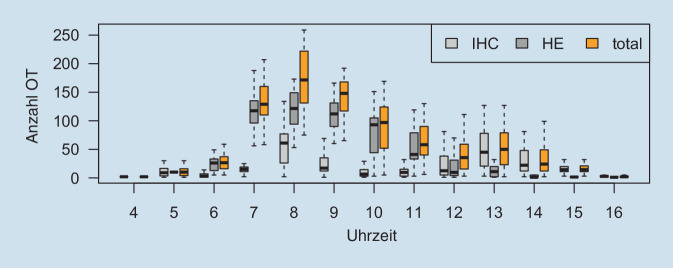

Das Institut für Pathologie der TU München produziert ca. 200.000 OT pro Jahr. Die Turnaround Time (TAT) von der Einsendung des Gewebes bis zur Befunderstellung beträgt in der Regel 24 h (Zuschnitt am selben Tag des Eingangs, entwässern über Nacht, einbetten, schneiden, färben und befunden am nächsten Tag). Diese TAT sollte durch die Einführung der Digitalisierung nicht verlängert werden, d. h., ein Scannen über Nacht, wie in vielen Laboren praktiziert, wurde bei der Bedarfs- und Workflowplanung nicht in Erwägung gezogen. Stattdessen definierte man die Befundung der anfallenden Fälle noch am gleichen Tag als unabdingbares Kriterium. In einer ersten Laboranalyse mittels LIS wurde zunächst die Anzahl und der Erstellungszeitraum der OT über den Tag gemessen, um Spitzenzeiten erkennen und die daraus resultierende benötigte Kapazität der Scanner erfassen zu können. In dieser Analyse konnten in der Spitze die Herstellung von 200 OT (Routine- und Immunlabor) pro Stunde identifiziert werden (Abb. 2).

### Implementierung des Scanvorgangs an bereits befundeten Präparaten

**Figure 3:**
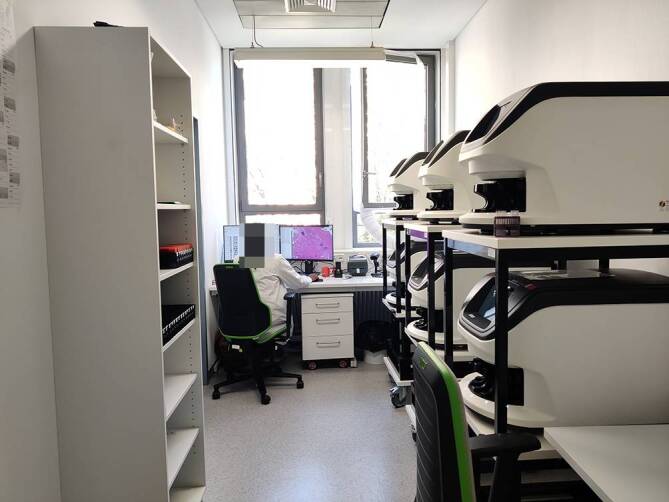

Die Implementierung der digitalen Pathologie wurde im Jahr 2021 mit 2 Scannern begonnen (Aperio GT450Dx, Leica Biosystems, Nußloch, Deutschland). Zunächst wurden retrospektiv nur OT aus dem Archiv digitalisiert, um erste Erfahrungen mit verschiedenen Scannern und der IT-Infrastruktur zu sammeln und die aktuelle Fallbefundung nicht durch mangelnde Erfahrung und technische Probleme zu belasten. In dieser Zeit wurden Schnittstellen zwischen Scannern und unserem LIS (Nexus Pathologie, Nexus AG, Villingen-Schwenningen, Deutschland) definiert und implementiert. Auch wurde der Scanningdurchsatz verschiedener Modelle (Hamamatsu NanoZoomer S60, Hamamatsu Photonics, Hamamatsu, Japan; 3DHistech P1000, 3DHISTECH Ltd., Budapest, Ungarn; Aperio GT450Dx) gemessen, um abschätzen zu können, wie viele Scanner und digitaler Speicherplatz zusätzlich benötigt werden. Alle getesteten Modelle lieferten hervorragende Bildqualität und kurze Scanningzeit. Wir entschieden uns hauptsächlich wegen kompakter Bauweise, LIS-Anbindung und diagnostischer Zulassung für das Leica-Modell. Ein Test verschiedener Modelle empfehlen wir allen Laboren, da die örtlichen Gegebenheiten und Ansprüche höchst individuell sind. Die Testphase war unerlässlich, um auch die örtliche/räumliche Integration der Scanner zu eruieren und so optimierte Laufwege für den besten Standort der Scanner zu finden. Die Wahl fiel aus unterschiedlichen Gründen (Platzmangel im Hauptlabor, vorhandene Anschlussbuchsen und IT-Infrastruktur sowie Ausbaumöglichkeiten) auf einen separaten Scanningraum (Abb. 3).

### Slide-Viewing-Software

Um die digitalen OT zu visualisieren, wurde ein institutseigener Slideviewer eingerichtet [19], der direkt an die Image-Management-Software der Scanner, die Speicherserver und das LIS angebunden ist. So können die digitalen Bilder der gescannten Fälle im LIS direkt aufgerufen und geöffnet werden. Diese Konstellation war nötig, um individuelle Anforderungen an den Viewer zu erfüllen, die nicht in Standardsoftware enthalten ist. So ermöglicht unser Viewer, interaktiv Rescan-Aufträge zu erteilen, wenn ein digitaler OT beispielsweise zu unscharf ist. Weiterhin können einzelne digitale Bilder zu Ausbildungszwecken anonymisiert heruntergeladen werden. Zudem ist eigene Forschung am effizienten Streamen von digitalen Slides mit unserer Software möglich [20, 21].

### Einrichtung eines Scanningteams

Um das Laborpersonal durch die zusätzlichen und neuen Arbeitsschritte zunächst nicht übermäßig zu belasten und damit eventuell Aversionen gerne die Einführung des digitalen Workflows zu generieren/verstärken, wurde ein Scanningteam eingerichtet, das aktuell aus einer in Vollzeit angestellten technischen Mitarbeiterin sowie mehreren studentischen Hilfskräften besteht, die urlaubs-, krankheits- und arbeitsaufkommensadaptiert flexibel den Scanningprozess unterstützen. Ihre Kernaufgaben sind:

- Beaufsichtigen und Durchführen des Scanningprozesses,
- Annahme der gefärbten, gedeckelten und getrockneten OT aus dem Labor,
- vorbereiten der OT (kontrollieren auf Verunreinigungen und des Deckglases, ggf. Nachdruck eines unleserlichen Barcodes),
- beladen und entladen der Scanner,
- nachbereiten der OT (mit einem Marker als „gescannt“ markieren),
- Kontrolle der fertigen Scans (Reagieren auf Qualitätsrückmeldungen am Scanner, stichprobenartige Überprüfung auf Scanartefakte),
- Abgabe der OT an das Labor (zur Auslieferung bzw. Archivierung),
- koordinieren der technischen Anforderungen (Hardwarewartung, Softwareupdates, Reparaturen),
- verwalten von Accounts im Image-Management-System,
- dokumentieren von Qualitätskontrollen,
- dokumentieren und reagieren auf vom ärztlichen Personal rückgemeldete Fehler.

### Umstellung des Workflows im histopathologischen Eingangslabor

Ende 2022 erfolgte in unserem Institut die Initialisierung des Umstellungsprozesses auf prospektives Scannen des aktuellen Routineeingangs. Dies erfolgte zunächst in Form wiederkehrender, wöchentlicher Planungstreffen, an denen neben einem berufenen Professor für Computational Pathology, die ärztliche und technische Leitung des Routinelabors sowie das Scanningteam teilnahmen. Hier wurden die einzelnen Umstellungen diskutiert, danach im Labor implementiert und im darauffolgenden Treffen eine Woche später bewertet und ggf. adaptiert.

**Figure 4:**
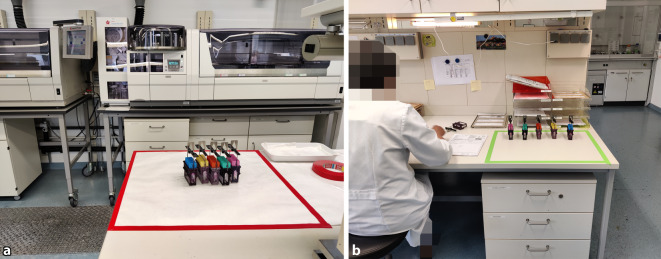

**Figure 5:**
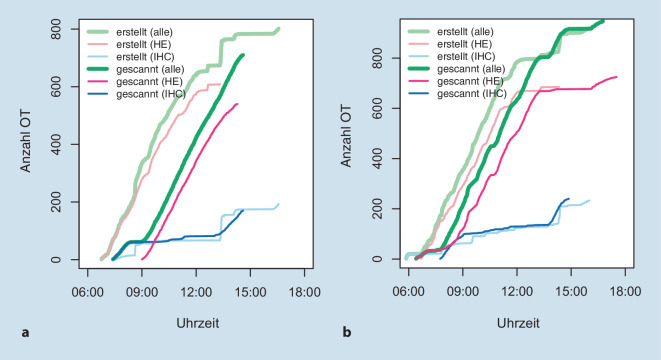

Durch die für den Scanvorgang notwendig gewordene Bereitstellung der bereits gefärbten OT für die Mitarbeiter des Scanningteams ergaben sich neue Laufwege. Die neue Schnittstelle zwischen Labor und Scanningteam wurde durch farblich markierte Ablageplätze für ungescannte und bereits gescannte Schnitte in rot und grün markiert (Abb. 4). Das prospektive Scannen implementierten wir zunächst mit kleinen, gut charakterisierten Schnittkollektiven (z. B. gastrointestinale Biopsien). Diese Schnittkollektive wurden mit farblich verschiedenen OT versehen und waren somit vom Labor leicht zu identifizieren (siehe Ständer [Racks] in Abb. 4). Hergestellte und gescannte OT wurden jeweils pro Tag gemonitort, die Differenz beim Folgetreffen vorgestellt und diese dann durch schrittweise Hinzunahme weiterer farblich codierter Schnittkollektive (z. B. Mammastanzen, Prostatastanze) für das prospektive Scannen Woche für Woche sukzessive verkleinert (Abb. 5). Mit dieser Art der Datenerhebung konnten auch zeitliche Engpässe erkannt und Maßnahmen frühzeitig ergriffen werden.

Auf Grundlage dieser Erkenntnisse erfolgte dann die Modifikation der bereits eingeführten Maßnahmen bzw. die Implementierung weiterer Schritte.

Eine der größten Herausforderungen stellte der zusätzliche Zeitbedarf an, der zum Scannen benötigt wird. Hier kamen 2 Aspekte zum Tragen: Zum einen bedarf es einer möglichst kurzen Scanzeit pro OT, zum anderen der Vermeidung von Verzögerungen durch Engpässe bei ineffizienter Beladung der einzelnen Scanner. Zwar wird jeder OT innerhalb von 2–3 min gescannt (je nach Gewebemenge auf dem OT), aber ein volles Rack mit 20 OT benötigt somit bereits 40–60 min und zu Spitzenzeiten sind mehrere Racks pro Scanner in der Warteschlange. Dadurch verlängert sich die Arbeitszeit des Laborpersonals und die Bereitstellung der OT an die PathologInnen verzögert sich.

**Figure 6:**
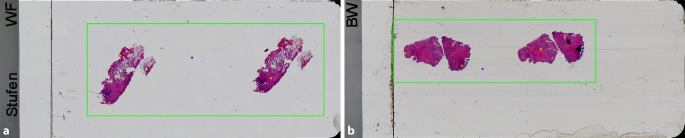

Diese Verzögerung konnte durch verschiedene Maßnahmen minimiert werden. Zunächst wurden weitere Scanner angeschafft (insgesamt aktuell 6 Scanner, 2 weitere bestellt). Weiterhin wurde der morgendliche Arbeitsbeginn der technischen AssistentInnen teil- und schrittweise um 30 bis 60 min nach vorne verlegt und die verschiedenen Arbeitsschichten im Labor zeitlich weiter auseinandergezogen. Zudem erfolgte auch eine Durchführung des Scanvorgangs bei eiligen Fällen und kleinen Biopsien auch bei halbvollen Racks am frühen Morgen, um möglichst bis zum Vorliegen der OTs mit größeren Gewebeproben später am Morgen keine Warteschleife zu erzeugen und um einen möglichst kontinuierlichen Arbeitsablauf zu erreichen. Eine weitere Maßnahme zur Effizienzsteigerung des Scanvorgangs umfasste eine Nachschulung des Laborpersonals zum Aufbringen der im Wasserbad befindlichen Paraffinschnitte auf die OT: Durch engeres und lineares Platzieren der Paraffinschnitte konnte eine deutliche Reduktion der zu scannenden Fläche (sog. Bounding Box, Abb. 6) erreicht werden, was wiederum zu deutlich kürzen Scanningzeiten führte. Auch wurde nochmals die Anzahl der aufgebrachten Stufen im Hinblick auf Leitlinien und Laborstandards überprüft und optimiert.

**Figure 7:**
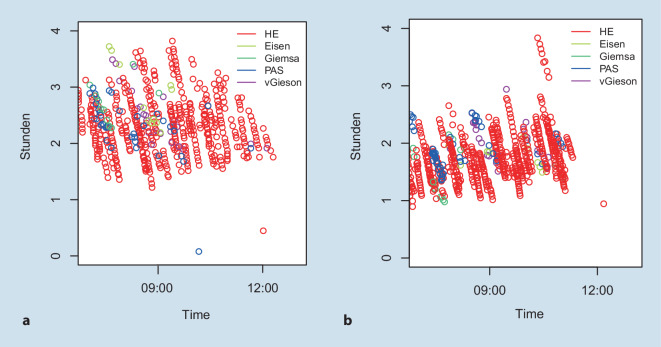

So konnte innerhalb von 6 Monaten die Gesamtzeit für die vollständige prospektive digitale Erfassung aller neuer OTs eines Tages von initial 3 h auf 0,5–1 h Verzögerung reduziert werden im Vergleich zur Ausgabe des vollständig ungescannten histopathologischen Schnitteingangs (Abb. 7).

### Herausforderungen und Dos and Don’ts

Die größten Herausforderungen, die in der Implementierung des digitalen Workflows beobachtet wurden, waren

- technische Probleme mit den Scannern durch nicht erkannte Slides, nicht erkannte Racks, unterbrochene Kommunikation mit dem LIS oder vollgelaufenen internen Speichern,
- Qualitätsprobleme der OT: zu viel Material auf OT, unlesbarer oder falscher Barcode, fehlendes oder falsch aufgebrachtes Deckglas, festklebende OT im Rack,
- Fehler im Betriebsablauf durch unzureichende Schulung des technischen Personals.

Die daraus resultierenden Maßnahmen umfassten folgende Punkte:

- Fehler am Scanner umgehend dem Anbieter melden.
- „Eingangscheck“ der zu scannenden OT: OT trocken? Barcode lesbar? Deckglas korrekt aufgebracht? Diese Maßnahmen kosten einige Sekunden, sparen aber dutzende Minuten im Falle eines Fehlers bzw. im Falle überstehender Deckgläser auch die Gefahr einer Beschädigung der Scannerlinsen.
- Regelmäßige Wartung der Scanner wie vom Hersteller empfohlen: Die hochpräzisen Instrumente sind anfällig für Staub, Schmutz, Wachs- und Kleberreste sowie für zu hohe Temperatur, ggf. wird die Implementierung eines externen Kühlsystems (Ventilator, Klimaanlage) im Raum notwendig.
- Frühe Einbeziehung des technischen Laborleitungspersonals.
- Regelmäßige Treffen während des Umstellungsprozesses. Kommunikation ist wichtig!
- Sorgfältige Planung des Laborbedarfs mit anfänglich etwas größerem Puffer und ggf. zeitweise Aufstockung des Personalschlüssels.
- Um die Bearbeitungszeit für das Scannen und die Befunderstellung am gleichen Tag konstant zu halten, bedarf es in Ausnahmefällen, z. B. bei hoher Arbeitsbelastung (zu viele Objektträger/zu wenig Laborpersonal) einer Anpassung des Workflows mit Ausgliederung einzelner Schnittkollektive, die dann an die ÄrztInnen ausgegeben, aber erst nachträglich gescannt werden.
- Einführung von Messtools wie Scanzeiten und anderer Metriken, um Änderungen im Workflow objektiv beurteilen zu können. Dazu bieten sich Slide-Tracking-Tools aus dem LIS an.

## Diskussion

Die Implementierung der digitalen Pathologie in den Laboralltag benötigt Zeit und Fingerspitzengefühl. Da von den technischen AssistentInnen zumindest in der Anfangs- und Übergangszeit bis zur vollständigen Digitalisierung eine Umstellung des in der Regel schon langjährig etablierten und eingespielten Workflows erwartet wird sowie auch eine Mehrbelastung durch Einführung neuer/zusätzlicher Aufgaben sehr wahrscheinlich ist, empfiehlt es sich, bereits früh die technischen Führungskräfte und technisch interessierte bzw. veränderungswillige Mitarbeiterinnen im histopathologischen Eingangslabor in die Umsetzung miteinzubeziehen und, soweit möglich, auch auf deren Kritik und Bedürfnisse einzugehen. Dies geht unserer Erfahrung nach mit einem zumindest zeitweisen Personalmehrbedarf einher, der aber Aversionen gegen die digitale Pathologie und Ängste, z. B. vor nicht adäquat kompensierter Mehrarbeit oder drohendem Arbeitsplatzverlust, minimierte und so die Einführung deutlich erleichterte. Dieser Personalmehrbedarf kalkulierte sich bei uns mit etwa 200.000 OT/Jahr auf etwa 1–2 Mitarbeiter (wobei dies nicht zwingend technische Mitarbeiter sein müssen) für Scannen, (Krankheits‑/Urlaubs‑)Vertretung und Arbeitsmehraufwand im Labor. Urlaubspläne und Regelungen für den Überstundenausgleich müssen ggf. überarbeitet werden. Bei der Implementierung sollte auch der Planung der Laufwege große Bedeutung beigemessen werden, da diese als potenzieller Verzögerungsfaktor nicht zu unterschätzen sind.

Unsere Probleme haben sich hauptsächlich durch eine *fließende Übergangszeit* und einen *hybriden Digitalisierungsansatz* gelöst. In der *Übergangszeit *hatte das Personal nicht nur genug Zeit, sich an die neuen Arbeitsabläufe zu gewöhnen, sondern sie auch effizient und optimal in die vorhandenen Laborprozesse zu integrieren und selbst mitzugestalten. Die Scanner- und LIS-Hersteller hatten ebenfalls genug Zeit, um die Verbindung zwischen den Systemen zu implementieren und anfängliche Hard- und Softwareprobleme zu beseitigen. Insbesondere musste der Hersteller unseres langjährig etablierten LIS neue Schnittstellen implementieren, um digitale Pathologie zu unterstützen. Im *hybriden Ansatz* werden die OT nach dem Scannen weiterhin an die PathologInnen ausgeliefert. Dies ist zwar etwas personalintensiver und arbeitsaufwendiger, allerdings in der Übergangsphase unerlässlich, da vollständige digitale Befundung noch nicht möglich und von vielen ÄrztInnen anfänglich auch nicht vollständig gewünscht ist. Ein hybrider Ansatz ist zudem fehlertolerant und ermöglicht den direkten Vergleich zwischen physischen OT und digitalen Bildern. In einem ausschließlich digitalen Labor hingegen werden die OT nach dem Scan direkt archiviert und die Befundung erfolgt ausschließlich digital. Das entlastet das Laborpersonal, da die Archivierung nicht zeitkritisch ist und in andere Zeiträume ausgelagert werden kann. Sollten die OT trotzdem nötig sein, können sie vom Viewer aus direkt geordert werden. Hierzu ist eine Validierung der digitalen Pathologie im Labor nötig, wie sie z. B. vom College of American Pathologists [22], dem Royal College of Pathologists [23], oder dem Bundesverband deutscher Pathologen [24] empfohlen wird.

## Fazit für die Praxis

- Die Implementierung der digitalen Pathologie ist zumindest in der Anfangszeit kosten- und personalintensiv.
- Die meisten Kosten nivellieren sich über die Zeit durch die daraus entstehenden unschätzbaren Vorteile wie flexibles und effizienteres Arbeiten, Talentakquise über die örtlichen Grenzen hinaus oder KI-gestützte Analysen als Tool zur optimierten Befundung und Maßnahme gegen die immanenter werdende Lücke an FachärztInnen für Pathologie.
- Ein sorgfältiges Changemanagement ist im Personalbereich unabdingbar, da auch sensible Bereiche wie Arbeitszeit, Urlaubs- und Überstundenmanagement gegebenenfalls überarbeitet werden müssen.
